# circPDK1 competitively binds miR-4731-5p to mediate GIGYF1 expression and increase paclitaxel sensitivity in non-small cell lung cancer

**DOI:** 10.1007/s12672-024-01003-2

**Published:** 2024-05-11

**Authors:** YunYin Feng, TaoLong Zhang, Hong Liu

**Affiliations:** 1 Department of Respiratory, Kaihua County Traditional Chinese Medicine Hospital, No.10 Zhongshan Road, Qinyang Office, Quzhou City, 324000 Zhejiang Province China; 2 Department of Gastroenterology, Kaihua County Traditional Chinese Medicine Hospital, Quzhou City, 324300 Zhejiang Province China

**Keywords:** Non-small cell lung cancer, circPDK1, miR-4731-5p, GIGYF1, Paclitaxel

## Abstract

**Objective:**

To investigate the action of circPDK1 in paclitaxel (PTX) resistance in non-small cell lung cancer (NSCLC).

**Methods:**

circPDK1, miR-4731-5p, and GIGYF1 levels were determined by RT-qPCR and Western blot. Cell proliferation was detected by CCK-8 and colony formation assay, apoptosis by flow cytometry, invasion by Transwell assay. The targeting relationship between miR-4731-5p and circPDK1 or GIGYF1 was confirmed by dual luciferase reporter gene and RIP assay. A xenograft tumor model was established to determine the role of circPDK1 in PTX resistance.

**Results:**

circPDK1 was overexpressed in PTX-resistant NSCLC, and depleting circPDK1 hampered proliferation and invasion of PTX-resistant cells, activated apoptosis, and improved PTX sensitivity. circPDK1 bound to miR-4731-5p, and increasing miR-4731-5p expression salvaged the effect of circPDK1 depletion on PTX resistance. miR-4731-5p directly targeted GIGYF1, and upregulating GIGYF1 offset the promoting effect of circPDK1 knockdown on PTX sensitivity. NSCLC tumor growth was inhibited and PTX sensitivity improved when circPDK1 was suppressed.

**Conclusion:**

Depleting circPDK1 promotes PTX sensitivity of NSCLC cells via miR-4731-5p/GIGYF1 axis, thereby inhibiting NSCLC pregnancy.

## Introduction

About 80% of all lung cancers are non-small cell lung cancer (NSCLC), mainly adenocarcinoma and squamous cell carcinoma [1], with 5-year survival rates of 10–15% [2]. Currently, NSCLC patients are diagnosed at stages IIIB or IV due to the absence of effective diagnostic measures and biomarkers [3], with distant metastasis and poor prognosis. Patients with advanced NSCLC benefit from chemotherapy, but their overall survival rate is less than 20% [4]. In NSCLC, tumor metastasis is thought to be the underlying cause of death. Combined chemotherapy with radiotherapy is the standard and conservative treatment strategy for patients with advanced NSCLC without pleural or pericardial effusion, such as paclitaxel (PTX) and platinum [5]. However, drug resistance has hindered the effect of chemotherapy, and new clinical treatment methods need to be developed urgently.

Microtubule inhibitor PTX is a diterpenoid alkaloid with anti-tumor properties that induces apoptosis by inhibiting spindle microtubule movement [6–8], thereby blocking G2/M cells and inhibiting cell proliferation [9]. PTX has been applied in the clinical treatment of malignant tumors, but its anti-tumor activity is limited by various factors, such as β-tubulin overexpression, mitotic checkpoint change, and ABC transport protein, thereby developing primary or secondary drug resistance [10–12]. Therefore, new strategies for treating NSCLC must be developed as a result of further study into the molecular mechanisms of PTX resistance in NSCLC.

circRNAs are endogenous RNAs [13] that are involved in disease progression [14–16] and different biological processes in cancers [17] and are key actors in tumor carcinogenesis and drug resistance [18, 19]. circPDK1 is transcribed from the pyruvate dehydrogenase kinase 1 (PDK1) gene, the function of which has never before been identified in NSCLC. circRNAs can exert their biological activity as competing endogenous RNAs (ceRNAs) for microRNAs (miRNAs), which regulate tumor development by participating in drug resistance. Whereas circPDK1 regulates cell proliferation, apoptosis, invasion and migration in lung squamous cell carcinoma, renal cancer and colon cancer through competitive adsorption of miRNAs. In NSCLC, hsa-circ-0030998 reduces PTX resistance by sponging miR-558 [20], and hsa-circ-0002483 enhances PTX sensitivity by regulating miR-182-5p [21]. Several other circRNAs, such as circ-0076305 and circ-0004015, have also been shown to be strongly associated with resistance [22, 23]. circPDK1 is upregulated in human lung cancer based on microarray data. However, there is still a lack of understanding as to how circPDK1 affects PTX resistance in NSCLC.

MiRNAs are small ncRNA molecules [24] that can target genes and have been implicated in tumor pathogenesis [25]. miR-4731-5p has tumor suppressor activity and can distinguish tumor stages with high specificity and sensitivity [26]. It is recorded that miR-4731-5p inhibits gliomas and is lowly expressed cancer [27], such as in oral lichen planus and glioblastoma [28]. MiR-4731-5p levels are consistently down-regulated in both NSCLC tissues and cells, and its up-regulation inhibits NSCLC cell viability, invasion and EMT and promotes apoptosis. However, it is not known whether miR-4731-5p modulates NSCLC PTX resistance. GIGYF1 is a member of the GYF adaptor protein family. GIGYF1 binds to growth factor receptor binding 10 (Grb10), which binds to the activated insulin receptor and insulin-like growth factor-1 receptor, thereby negatively regulating receptor signaling, metabolic responses, and IGF1-induced mitosis. GIGYF1 plays a catalytic role in cancer progression, such as gastric cancer and malignant glioma [29]. However, whether GIGYF1 can be directly targeted by miR-4731-5p and its role in PTX resistance in NSCLC has not been investigated.

Since circPDK1 has a binding sequence of miR-4731-5p and GIGYF1, it suggests that circPDK1 may bind miR-4731-5p to regulate GIGYF1. As a result, this study aims to investigate how circPDK1/miR-4731-5p/GIGYF1 axis affects carcinogenesis and PTX resistance in NSCLC.

## Materials and methods

### Clinical samples

After surgical resection, NSCLC tissue samples (n = 40) and normal tissue samples (n = 40) were collected from Kaihua County Traditional Chinese Medicine Hospital. The distance of normal lung tissue samples from the margin of cancer tissue was ≥ 2 cm, and the patient did not undergo any preoperative chemoradiotherapy. The clinical characteristics of the patients are shown in Table 1. According to the Response Evaluation Criteria in Solid Tumors criteria, PTX-sensitive tumors were defined as those in which a ≥ 30% reduction in the largest lesion was observed, whereas PTX-resistant tumors were those that did not show any significant change after PTX treatment. All specimens were frozen in liquid nitrogen and kept at −80 ℃. This study obtained patients’ informed consent and was approved by the Research Ethics Committee of Kaihua County Traditional Chinese Medicine Hospital (No. 2019Z0611).

**Table 1:** Correlation between circPDK1 level and pathological indexes of NSCLC patients

Clinicopathologic features	Number of cases	circPDK1 expression		*p*-value
		Low (n = 20)	High (n = 20)	
Age (years)				0.525
≤ 60	18	8	10	
> 60	22	12	10	
Gender				0.519
Male	27	14	13	
Female	13	6	7	
Smoking status				0.2355
Ever	30	14	16	
Never	10	6	4	
Tumor size				0.010*
< 5 cm	15	11	4	
≥ 5 cm	25	9	16	
TNM stage				0.057
I–II	16	7	9	
III–IV	24	13	11	
Lymph node metastasis				0.043*
Yes	21	6	15	
No	19	14	5	
Tumor recurrence				0.0318*
Yes	25	8	15	
No	15	12	3	
Chemosensitivity				0.0425*
Yes	17	15	2	
No	23	5	18	

* Indicates significant difference

### Cell lines

Primary human bronchial epithelial cells (HBE) and NSCLC cell lines (H1299, A549, H-125 and NCI-H292) were provided by Procell (Wuhan, China). HBE cells were cultured in DMEM (Procell) containing 10% FBS (Procell) and 1% penicillin/streptomycin (Sigma-Aldrich) with 5% CO_2_ and 95% air. H1299 and A549 cells were put in RPMI-1640 medium (GIBCOBRL, USA) + 2 mM glutamine, and H-125 and NCI-H292 in RPMI-1640 medium (Invitrogen, USA) + 10% FBS (Invitrogen) + 1% penicillin/streptomycin (Sigma-Aldrich). PTX (Sigma) was dissolved in DMSO (Sigma). The PTX-resistant H1299/PTX cell line was established by exposing H1299 cells to increasing concentrations of PTX (0.1–0.5 μM) until the ability of the cells to grow in the presence of PTX at the same rate as parental cells in the absence of the drug. H1299/PTX cells were cultured in DMEM/F12 medium supplemented with 10% FBS (containing 5 μM PTX, 100 U/mL penicillin, and 100 mg/mL streptomycin) and routinely incubated overnight (5% CO_2_, 37 °C) [30].

PTX-resistant A549/PTX cell line was established by exposing A549 cells to increasing concentrations of PTX (50 nM-250 nM). To maintain the PTX-resistant phenotype of A549/PTX cells, 0.1 μM PTX was added to the culture medium and routinely cultured overnight (5% CO_2_, 37 °C) [31].

### Cell transfection

Cell transfection was achieved in H1299/PTX cells and A549/PTX cells by Lipofectamine 2000 (Invitrogen). si-circPDK1, si-NC, miR-4731-5p mimics/inhibitor, miR-NC, pcDNA3.1-GIGYF1, and pcDNA3.1-vector were prepared by GenePharma (Shanghai, China). RT-qPCR was conducted to evaluate the transfection efficiency.

### RT-qPCR

Based on Trizol reagent (Thermo, USA), total RNA was extracted from NSCLC tissues and cells, of which the concentration was determined using BCA kits (Invitrogen). Then, miRNA cDNA was generated with miRNA reverse transcription kit (TaKaRa), and mRNA/circRNA cDNA with PrimeScript^™^RT Reagent kit (TaKaRa). Next, three repeats of RT-qPCR were completed for each sample on the CFX96 Contact real-time fluorescent quantitative PCR assay system (Bio-Rad, USA) using the SYBR Green PCR Premix kit (Invitrogen). RNA expressions were calculated using 2^−ΔΔCt^ and normalized to GAPDH or U6. The primers are shown in Table 2.

**Table 2:** Primer sequence

Gene	Forward primer (5′ → 3′)	Reverse primer (5′ → 3′)
circPDK1	GCATTACAACACCAACCACG	CGAGTGGTACGTGAGGGAC
GIGYF1	AGCTGCAGGACAAGGAGTTC	CCTCCTCAAAGCCACATCGT
miR-4731-5p	AGGCAGTGTATTGTTACGC	GCAGGGTCCGAGGTATTC
GAPDH	GTGGGCATCAAT GGATTTGG	ACACCATGTATTCCGG GTCAAT
U6	CTCGCTTCGGCAGCACATATACT	AACGCTTCACGAATTTGCGT

### CCK-8 analysis

H1299/PTX cells and A549/PTX cells were placed into 96-well plates at 2 × 10^3^ cells/well. After the specified time (0, 24, 48, and 72 h), 10 μL CCK-8 (Beyotime, Shanghai, China) was supplemented and reacted for 1 h before reading optical density at 450 nm on a microplate reader (Bio-Rad).

H1299/PTX cells and A549/PTX cells at logarithmic growth were placed in 96-well plates (1 × 10^4^ cells/well) after digestion and cultured for 24 h. Then, 0.05, 0.2, 0.8, 3.2, 12.8 μM PTX was added and reacted for 48 h. Afterward, cells were combined with 10 μL CCK-8 reagent at 37 ℃ for 2 h, and absorbance (450 nm) was read on a microplate reader (Bio-Rad). The growth curve was plotted using Graphpad Prism to calculate the IC_50_ value of PTX.

### Colony formation test

H1299/PTX cells and A549/PTX cells were plated in 6-well plates in an amount of 5 × 10^3^ cells per well and cultured for 36 h in 10% FBS-DMEM (37 ℃, 5% CO_2_). Then fresh medium + 2 μM PTX were replaced and cells were stimulated for 7 days. Cell culture was terminated upon the appearance of colonies. Then, cells were rinsed twice with PBS (Beyotime), fixed with 4% formaldehyde (Beyotime) for 10 min, stained with 5% crystal violet (Sigma) for 10 min, and calculated by microscopy (Olympus, Japan).

### Flow cytometry

Apoptosis rate was assessed by Annexin V-FITC/PI Apoptosis Detection kit (Invitrogen). H1299/PTX cells and A549/PTX cells (1 × 10^5^ cells) were suspended in a 1 × Binding Buffer (500 μl), further reacted with Annexin V-FITC (5 μl) and PI (5 μl) for 15 min without light, and detected on a FACScan^®^ flow cytometer (BD Biosciences, USA).

### Transwell experiment

The upper chamber of Transwell chamber (Costar, USA) was coated with Matrigel, in which 200 μL serum-free DMEM at cell concentration of 5 × 10^6^ cells/ml was added. Meanwhile, 600 μL 10% FBS-DMEM (HyClone) was added to the lower compartment and cultured for 24 h (37 ℃, 5%CO_2_). Next, non-invasive cells were removed, and the remaining cells after PBS treatment were fixed with 4% paraformaldehyde (Beyotime), treated with 0.5% crystal violet (Sinopharma), and observed by inverted microscopy (Olympus) in 5 fields.

### Dual luciferase reporter experiment

Circbase (http://circbase.org/) and TargetScan (www.targetscan.org) were utilized to predict the potential binding sites of miR-4731-5p, circPDK1 and GIGYF1. GenePharma synthesized wild-type or mutant circPDK1 fragments and GIGYF1 containing miR-4731-5p binding sites. The fragments were inserted into pmirGLO vector (Promega) to complete the construction of circPDK1-WT, GIGYF1-WT, circPDK1-MUT, and GIGYF1-MUT. H1299/PTX cells and A549/PTX cells were put in 96-well plates with 1 × 10^4^ cells/well and transfected with luciferase reporter and miR-4731-5p mimic or mimic-NC using Lipofectamine^®^3000 (Invitrogen) for 48 h. Assays of luciferase activity were conducted using a dual luciferase assay system (Promega).

### RIP experiment

RIP was determined using Magna RIP kit (Millipore). A lysate was produced by lysing H1299 cells and A549 cells with RIP lysis buffer and incubated with magnetic beads containing anti-AgO2 (Abcam, USA) or anti-IgG (Abcam) at 4 ℃ for 6 h. The immunoprecipitate bound to magnetic beads was eluted with elution buffer, and circPDK1, miR-4731-5p and GIGYF1 were analyzed by RT-qPCR after purification of RNA.

### Xenotransplantation of nude mice

Animal procedures were approved by the Kaihua County Traditional Chinese Medicine Hospital Animal Care and Use Committee (No. 201909ZJ41). Twenty 6-week-old BALB/c nude mice (Shanghai SLAC Laboratory Animals Co., Ltd., Shanghai, China) were randomly divided into si-NC group, si-NC + PTX group, si-circPDK1 group, and si-circPDK1 + PTX group. H1299 cells stably transfected with si-circPDK1 and si-NC were re-suspended in PBS buffer (1 × 10^5^ cells/ml), and nude mice were given 200 mL of H1299 cell suspension subcutaneously in the left armpit. A week later, 3 mg/kg PTX or PBS injections were administered intraperitoneally to mice every 3 days. In a pathogen-free environment at 22 ± 3 °C with 50 ± 15% relative humidity, 12 h of light/dark cycle were maintained for the mice. Every 7 days, tumor volume was measured and calculated using a caliper (short diameter^2^ × long diameter × 0.5). 35 days later, tumors were resected from euthanized mice.

### Immunohistochemistry

Tumors were fixed overnight in 10% formalin (Thermo Fisher Technology), embedded in paraffin, and sliced to 4 μm. After dewaxing with xylene (Beyotime), tissue sections were blocked by 0.3% H_2_O_2_ for 10 min and bound with PBS containing 0.3% Triton X-100 and 5% FBS for 1 h. Next, GIGYF1 antibody (ab121784, Abcam) was reacted at 4 °C overnight, as well as the secondary antibody (1:1000; ab6720, Abcam) for 1 h. Sections were developed with DAB (Vector Labs, USA) and re-stained with hematoxylin for 2 min, followed by microscopic imaging (Leica).

### Western blot

Tissues and cells were lysed on ice with RIPA lysis buffer (Vazyme, FD008) for 20 min, and protein concentrations were detected with Pierce BCA Protein Assay Kit (Rockford). The protein was isolated using 10% SDS-PAGE, transferred to a PVDF membrane (Millipore), sealed with 5% skim milk for 2 h, and treated with GIGYF1 (Abcam; ab121784) and GAPDH (Abcam; ab37168), Ki-67 (Abcam; ab270650), E-cadherin (Abcam; ab233611), N-cadherin (Abcam; ab254512) overnight at 4 °C, as well as secondary antibody (Abcam; ab205719) at 37 ℃ for 1 h. Results were developed with enhanced chemiluminescence detection kit (Vazyme; E411-04) and analyzed in the FluorChem^™^ system.

### Data analysis

Experimental data underwent statistical analysis based on SPSS20 statistical software. Measurement data were presented as ˉx ± s and tested by t-test or one-way ANOVA. *P* < 0.05 or < 0.01 indicated statistically significant differences.

## Results

### circPDK1 is highly expressed in PTX-resistant NSCLC

**Fig. 1:**
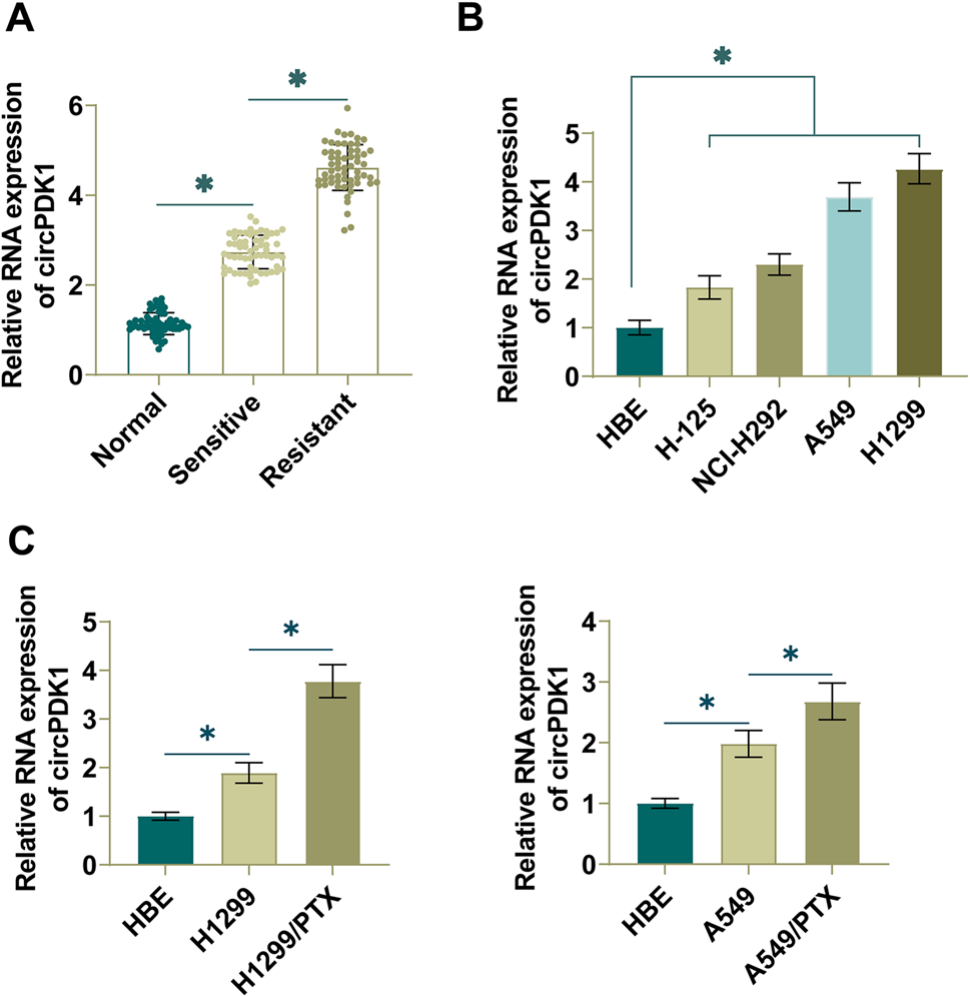
High expression of circPDK1 in NSCLC. **A** RT-qPCR measured circPDK1 in NSCLC tissues and normal tissues. **B** RT-qPCR measured circPDK1 in 4 NSCLC cell lines and HBE cells. **C** RT-qPCR measured circPDK1 in cells. *P < 0.05

To explore the role of circPDK1 in NSCLC progression, RT-qPCR technique detected circPDK1 levels in NSCLC and revealed an upregulation in circPDK1 expression in NSCLC tissues (Fig. 1A). High circPDK1 was also shown in NSCLC cells (H1299, A549, H-125, and NCI-H292), especially H1299 and A549 cells (Fig. 1B), so H1299 cells and A549 cells were selected for next experiments. The results by RT-qPCR showed that circPDK1 expression was elevated in H1299 and A549 cells, and the expression level was further increased in H1299/PTX and A549/PTX cells (Fig. 1C). In addition, clinicpathological parameters and circPDK1 expression levels were also analyzed in NSCLC patients. circPDK1 median value in NSCLC tissues was used as a cut-off value to distinguish high-expression group from low-expression group, and it was found that the increase of circPDK1 expression was significantly correlated with tumor size, late TNM, tumor recurrence, and drug sensitivity (Table 1). These data suggest that high expression of circPDK1 in NSCLC predicts a poor prognosis for NSCLC patients and may play a role in the development of NSCLC.

### Depleting circPDK1 enhances PTX sensitivity of NSCLC cells

**Fig. 2:**
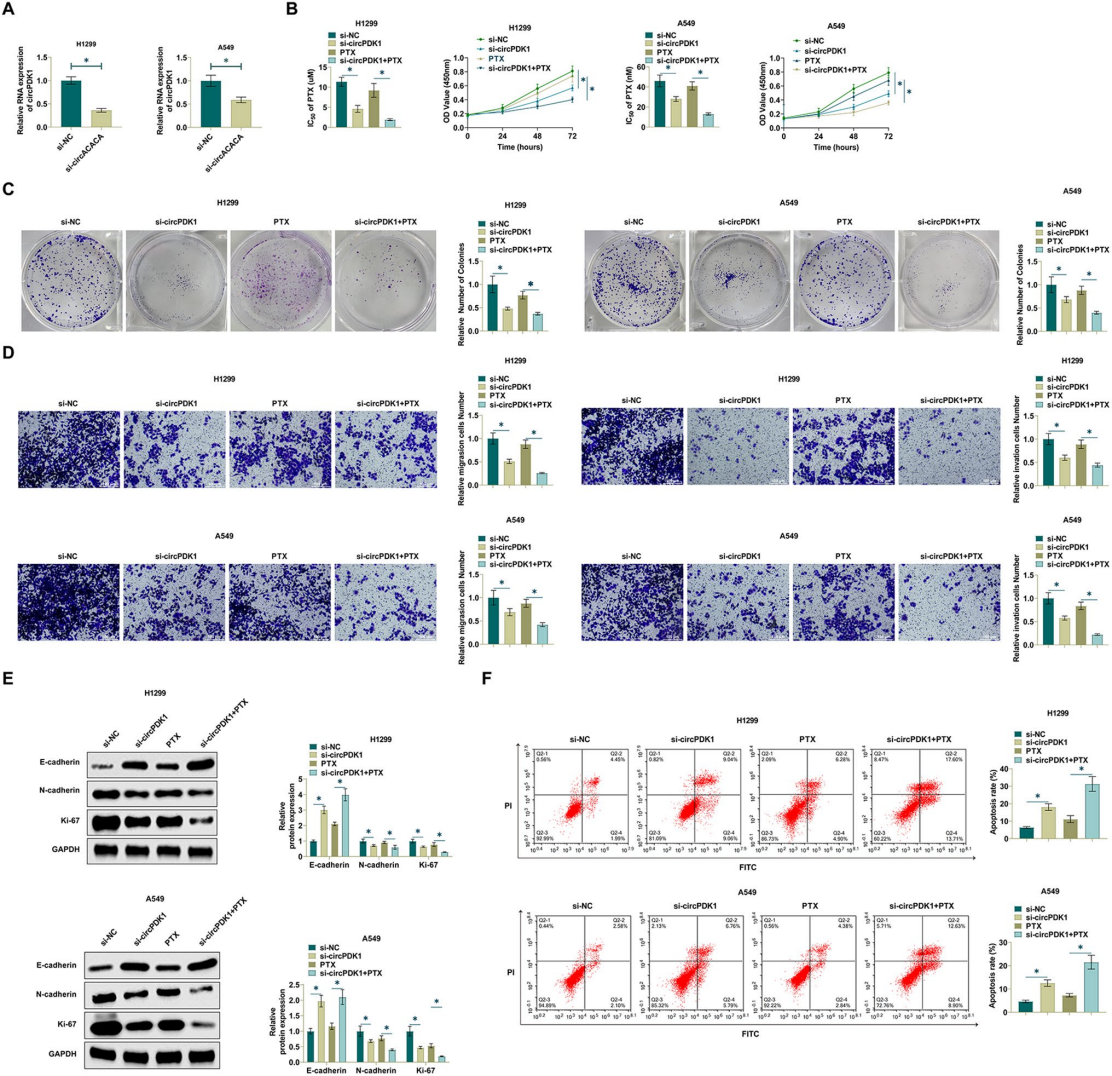
Knocking down circPDK1 enhances PTX sensitivity of NSCLC cells. Si-circPDK1 was transfected into H1299/PTX and A549/PTX cells. **A** RT-qPCR measured circPDK1. **B** CCK-8 method determined cell proliferation and PTX IC50 value. **C** Clone formation assay determined cell proliferation. **D** Transwell assay evaluated cell invasion. **E** Western Blot assay detected EMT-related proteins in cells. **F** Flow cytometry measured apoptosis rate. *P < 0.01

Loss-of-function assay assays were performed in PTX-resistant cells using siRNA targeting circPDK1 to assess the effect of circPDK1 on PTX resistance. Transfection of si-circPDK1 into H1299/PTX and A549/PTX cells to knock down circPDK1 expression. si-circPDK1 decreased circPDK1 expression in H1299/PTX cells and A549/PTX cells as observed by RT-qPCR (Fig. 2A). CCK-8 assay showed that si-circPDK1 significantly inhibited the proliferative ability of PTX-resistant cells and the IC50 of PTX, which indicated that si-circPDK1 inhibited the resistance of PTX-resistant cells to PTX. Therefore, silencing circPDK1 further enhanced the effect on the proliferation of PTX-resistant cells in the presence of PTX (Fig. 2B). Colony formation tested that silencing circPDK1 reduced the number of PTX-resistant cell colonies. Whereas, in the presence of PTX, enhanced the effect of silencing circPDK1 on the proliferation of PTX-resistant cells (Fig. 2C). The main reason why NSCLC is difficult to cure is that it is highly metastatic. Therefore, the effect of silencing circPDK1 on cell invasion ability was evaluated through Transwell assay. The number of migrating and invading cells was significantly reduced, and invasion ability was weakened due to the intervention of si-circPDK1. PTX promoted the effect of silencing circPDK1 on invasive capacity (Fig. 2D). To further explore the regulatory mechanism of si-circPDK1 on H1299/PTX cells, EMT-related proteins were determined by Western Blot. Silencing circPDK1 enhanced E-cadherin and hampered N-cadherin and Ki-67 expressions; The effect of silencing circPDK1 on EMT proteins was promoted after PTX action (Fig. 2E). Further, flow cytometry showed that suppressing circPDK1 significantly enhanced H1299/PTX and A549/PTX cell apoptosis; PTX further enhanced the role of si-circPDK1 (Fig. 2F). Taken together, this suggests that circPDK1 promotes the proliferation and migration of NSCLC/PTX cells, enhances PTX resistance, and participates in the regulation of NSCLC cell invasion through the EMT pathway, contributing to the development of NSCLC.

### circPDK1 binds miR-4731-5p in a competitive manner

**Fig. 3:**
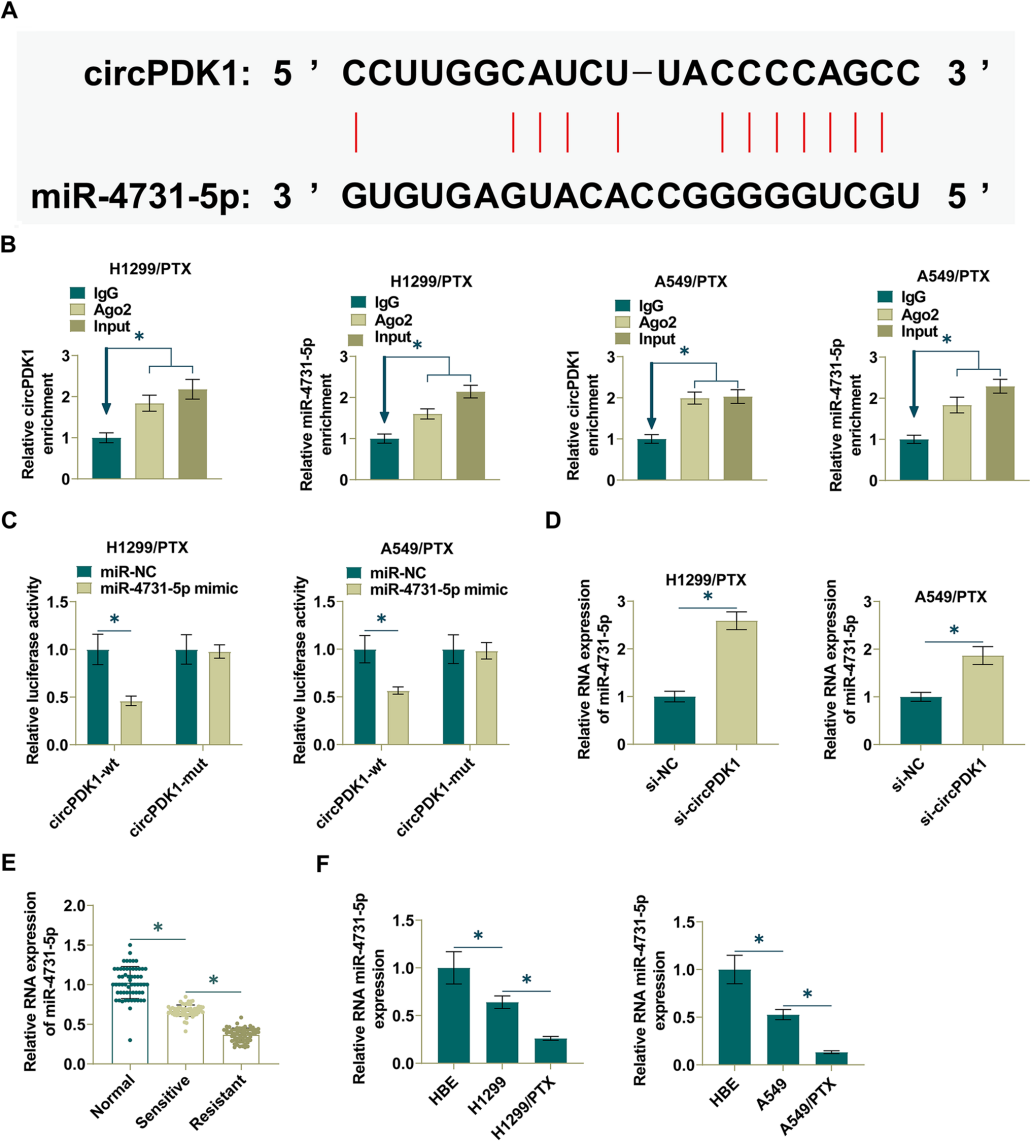
circPDK1 competitively binds miR-4731-5p. **A** Starbase predicted the binding sites of miR-4731-5p and circPDK1. **B** RIP tested circPDK1 and miR-4731-5p enrichment in H1299 and A549 cells. **C** Dual luciferase reporting assay detected the direct targeting relationship between miR-4731-5p and circPDK1 in H1299 and A549 cells. **D** RT-qPCR measured miR-4731-5p in H1299/PTX and A549/PTX cells transfected with si-circPDK1 or si-NC. **E** RT-qPCR measured miR-4731-5p in NSCLC tissues. **F** RT-qPCR measured miR-4731-5p in HBE, H1299, H1299/PTX, A549, and A549/PTX cells. *P < 0.01

To explore the molecular mechanisms of circPDK1 in NSCLC, potential target miRNAs of circPDK1 were identified through bioinformatics website starBase3.0 (http://starbase.sysu.edu.cn/), and miR-4731-5p was confirmed as the direct target. Potential binding sites for miR-4731-5p and circPDK1 were predicted (Fig. 3A). RIP detection implicated that circPDK1 and miR-4731-5p were immunoprecipitated by Ago2, suggesting that miR-4731-5p is the target of circPDK1 in NSCLC cells (Fig. 3B). To verify whether circPDK1 directly targets miR-4731-5p, a dual luciferase reporter assay was performed. The data confirmed that the luciferase activity decreased after co-transfecting miR-4731-5p mimic and WT-circPDK1. After co-transfecting miR-4731-5p mimic and Mut-circPDK1, luciferase activity did not change significantly (Fig. 3C). Further, depleting circPDK1 enhanced miR-4731-5p expression (Fig. 3D). Then, RT-qPCR observed that NSCLC tissues showed lower miR-4731-5p compared to normal tissues (Fig. 3E) and H1299/PTX and A549/PTX cells had lower levels than H1299 and A549 cells (Fig. 3F). These data suggest that circPDK1 directly targets miR-4731-5p in NSCLC.

### circPDK1 regulates PTX sensitivity by miR-4731-5p

**Fig. 4:**
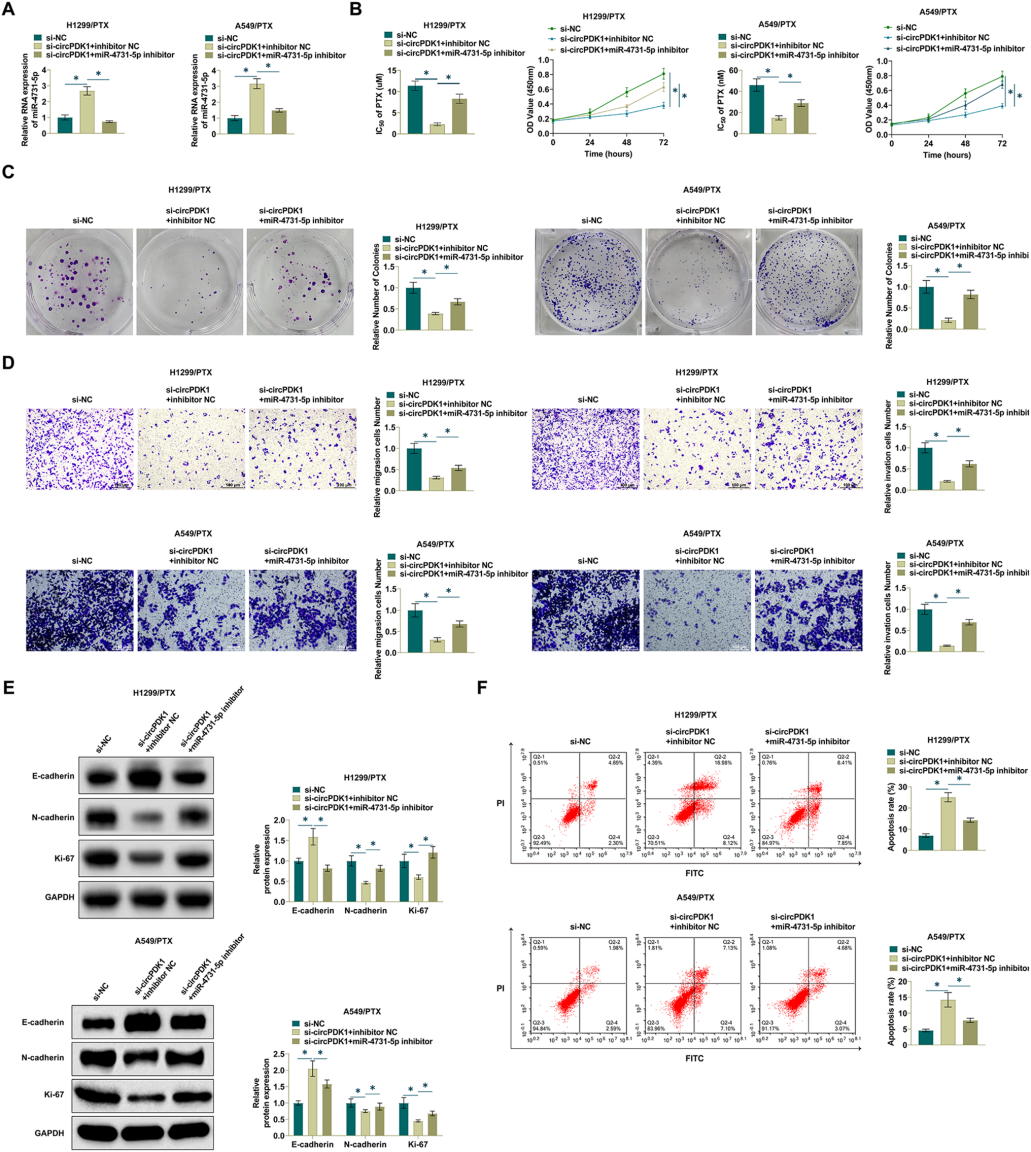
circPDK1 regulates PTX sensitivity by targeting miR-4731-5p. Si-circPDK1 and miR-4731-5p inhibitor were transfected into H1299/PTX and A549/PTX cells. **A** RT-qPCR measured miR-4731-5p. **B** CCK-8 method determined cell proliferation and PTX IC50 value. **C** Clone formation assay determined cell proliferation. **D** Transwell assay evaluated cell invasion. **E** Western Blot assay detected EMT-related proteins in H1299/PTX and A549/PTX cells. **F** Flow cytometry measured apoptosis rate. *P < 0.05

The interrelationship between circPDK1 and miR-4731-5p in NSCLC cells was explored by loss-of-function experiments to elucidate whether miR-4731-5p plays a role in circPDK1-mediated PTX resistance in NSCLC cells. si-circPDK1 was co-transfected with miR-4731-5p inhibitor in H1299/PTX and A549/PTX cells. Depleting circPDK1 increased miR-4731-5p expression, but this was reversed after transfecting miR-4731-5p inhibitor (Fig. 4A). Meanwhile, miR-4731-5p inhibition weakened the effect of si-circPDK1 on PTX-resistant cell proliferation, IC50 value for PTX, and colony number (Fig. 4B, C). Moreover, the impact of si-circPDK1 on cell invasion and migration was hindered by inhibiting miR-4731-5p (Fig. 4D). Meanwhile, suppressing miR-4731-5p prevented changes in E-cadherin, N-cadherin, and Ki-67 expression mediated by si-circPDK1 (Fig. 4E). Also, in H1299/PTX and A549/PTX cells, miR-4731-5p suppression diminished si-circPDK1's ability to promote apoptosis (Fig. 4F). Taken together, the results suggest that circPDK1 directly targets miR-4731-5p to regulate PTX sensitivity in NSCLC cells.

### miR-4731-5p targets GIGYF1

**Fig. 5:**
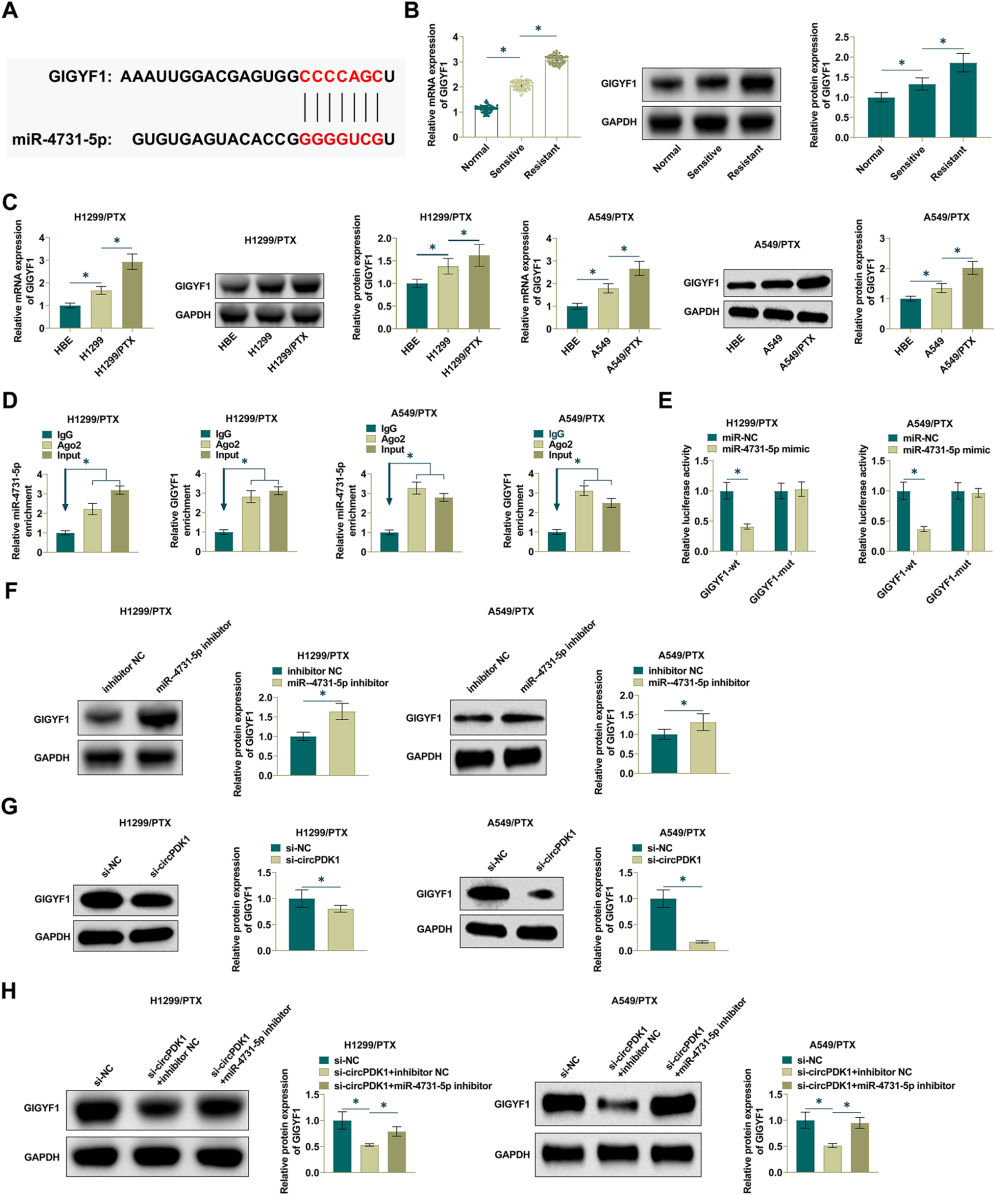
miR-4731-5p modulates GIGYF1. **A** Starbase predicted the binding site of miR-4731-5p and GIGYF1. **B** RT-qPCR and Western Blot measured GIGYF1 in NSCLC tissues. **C** RT-qPCR and Western Blot measured GIGYF1 in H1299/PTX and A549/PTX cells. **D** RIP determined the binding effect of miR-4731-5p and GIGYF1. **E** Dual luciferase reporting assay detected the targeting relationship between miR-4731-5p and GIGYF1. **F** Western Blot analysis of GIGYF1 protein in H1299/PTX and A549/PTX cells. **G** Western Blot measured GIGYF1 expression in H1299/PTX and A549/PTX cells. **H** Western Blot detected GIGYF1 expression in H1299/PTX and A549/PTX cells. *P < 0.05

The molecular target of miR-4731-5p was GIGYF1, which had a possible binding site with miR-4731-5p as predicted by Starbase bioinformatics website (Fig. 5A). Highly expressed GIGYF1 was found in both NSCLC tissues and H1299/PTX and A549/PTX cells (Fig. 5B, C). The interaction between GIGYF1 and miR-4731-5p was further explored and verified (Fig. 5D, E). Subsequently, the effect of miR-4731-5p on GIGYF1 expression was explored by Western Blot. miR-4731-5p inhibitor could promote GIGYF1 expression in PTX-resistant cells (Fig. 5F), while si-circPDK1 downregulated GIGYF1 expression (Fig. 5G). To further investigate the role of circPDK1 as a ceRNA mediating the regulation of GIGYF1 expression by miR-4731-5p, miR-4731-5p inhibitor was co-transfected with si-circPDK1 into H1299/PTX and A549/PTX cells, and the results showed that miR-4731-5p inhibitor reversed the silencing effect of circPDK1 on GIGYF1 expression (Fig. 5H). In summary, GIGYF1 is a target protein of miR-4731-5p and circPDK1 can act as a ceRNA adsorbing miR-4731-5p to upregulate GIGYF1 expression.

### Upregulating GIGYF1 prevents circPDK1 knockdown from exhibiting inhibitory effects on NSCLC

**Fig. 6:**
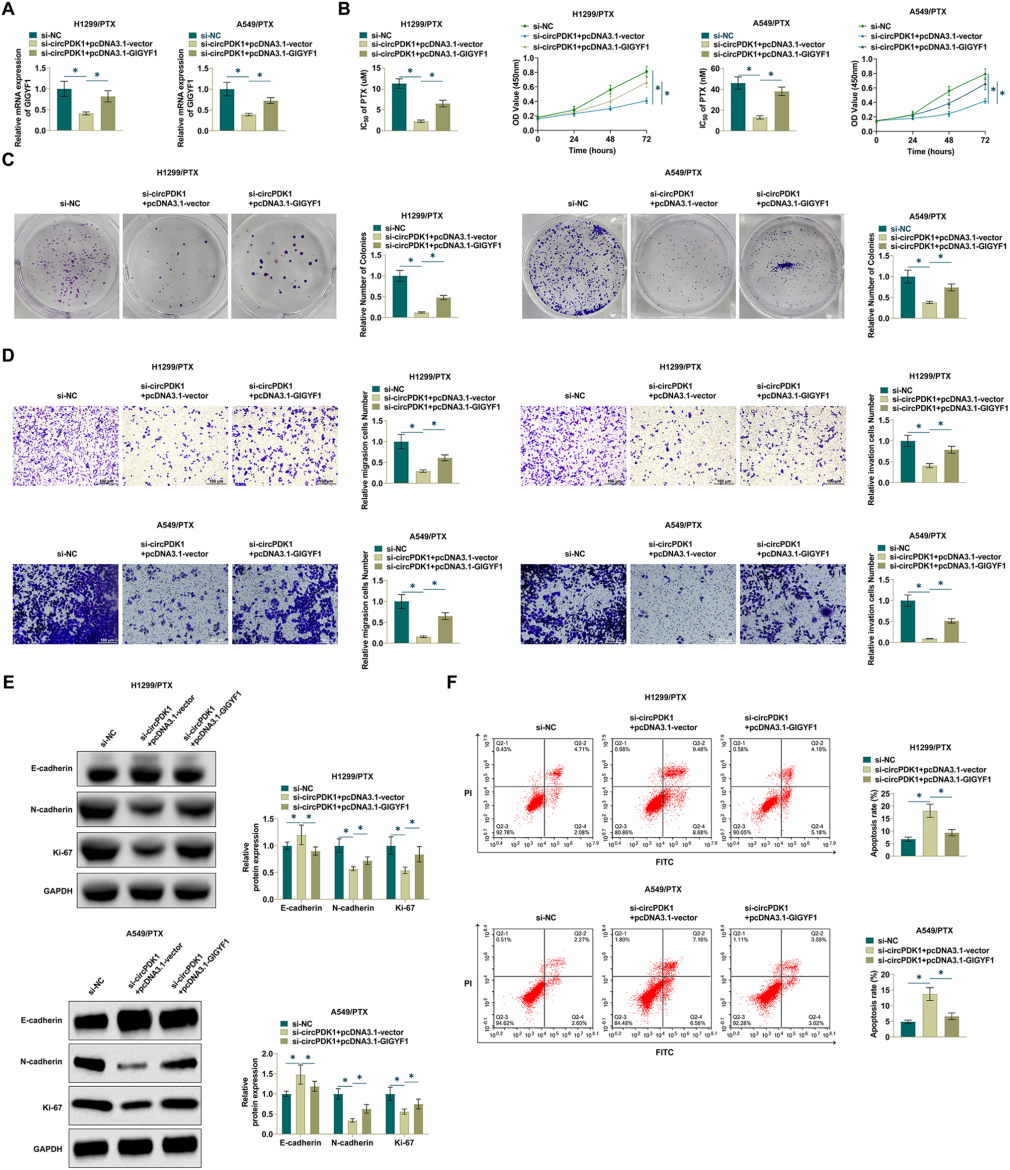
Upregulating GIGYF1 prevents circPDK1 knockdown from exhibiting inhibitory effects on NSCLC. si-circPDK1 and pcDNA 3.1-GIGYF1 were transfected into H1299/PTX and A549/PTX cells. **A** RT-qPCR measured GIGYF1. **B** CCK-8 method determined cell proliferation and PTX IC50 value. **C** Clone formation assay determined cell proliferation. **D** Transwell assay evaluated cell invasion. **E** Western Blot assay detected EMT-related proteins in H1299/PTX and A549/PTX cells. **F** Flow cytometry measured apoptosis rate. *P < 0.01

Knockdown of circPDK1 was further confirmed by loss-of-function experiments to inhibit the process of NSCLC drug resistance through the miR-4731-5p/GIGYF1 axis. After transfection of pcDNA3.1-GIGYF1 into H1299/PTX and A549/PTX cells, GIGYF1 was significantly increased as detected by RT-qPCR (Fig. 6A). si-circPDK1 and pcDNA 3.1-GIGYF1 were co-transfected into H1299/PTX and and A549/PTX cells. As measured, increasing GIGYF1 expression could impair the inhibitory effect of si-circPDK1 on cell proliferation and IC50 value for PTX (Fig. 6B, C). Increasing GIGYF1 expression reversed the suppressive effect of si-circPDK1 on cell invasion and migration ability (Fig. 6D). It was further found that pcDNA 3.1-GIGYF1 impeded the increase in E-cadherin expression and the decrease in N-cadherin and Ki-67 expression by silencing circPDK1 (Fig. 6E). Further, apoptosis rate of H1299/PTX and A549/PTX cells induced by silenced circPDK1 could be suppressed by overexpressing GIGYF1 (Fig. 6F). Taken together, circPDK1 regulates the miR-4731-5p /GIGYF1 axis to influence NSCLC progression.

### Suppressing circPDK1 inhibits tumor growth in vivo

**Fig. 7:**
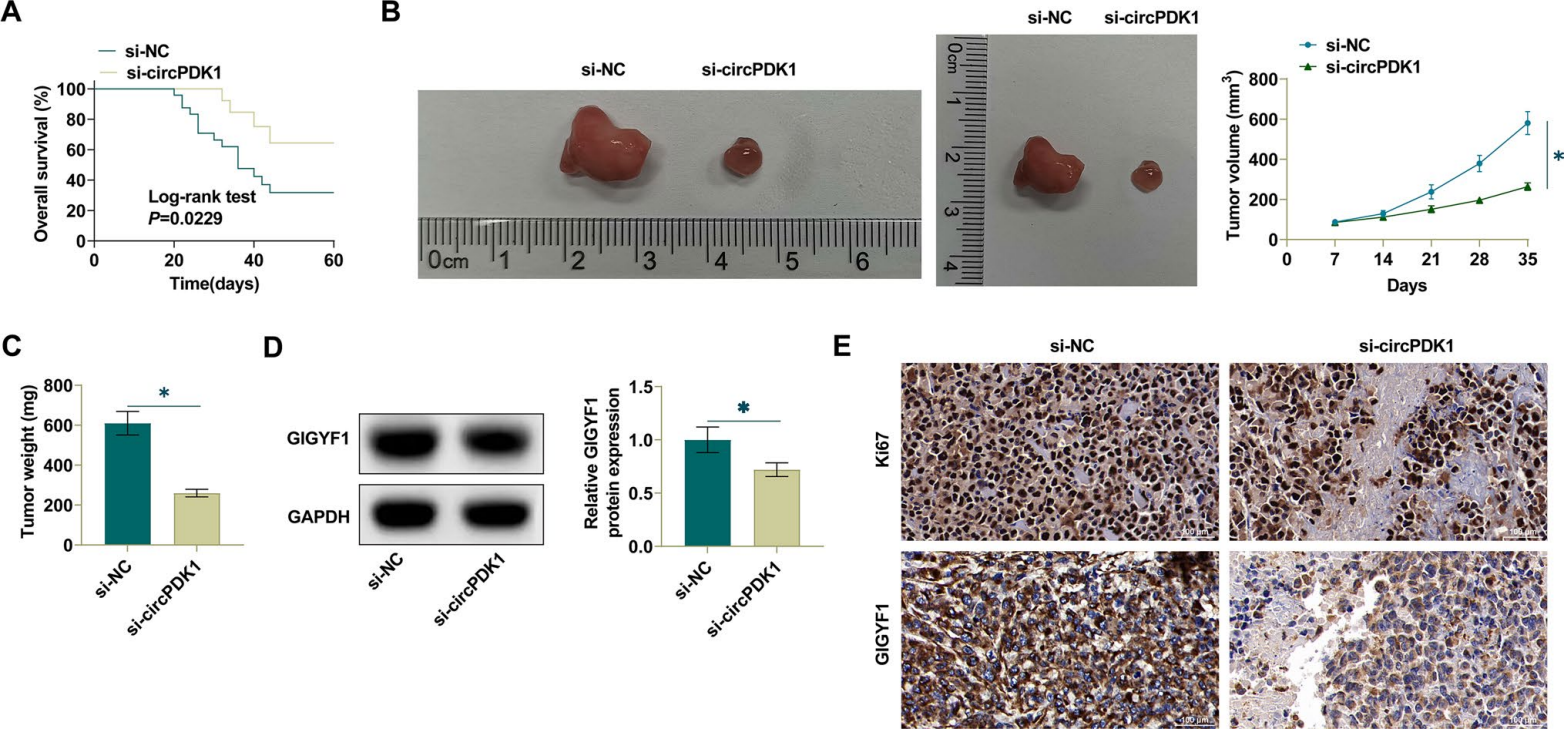
Suppressing circPDK1 inhibits tumor growth in vivo. **A** Survival curve analysis of xenograft NSCLC tumor-bearing mice with knockdown circUSP7 expression treated with PTX. **B** Tumor volume. **C** Tumor weight. **D** Western Blot analysis of GIGYF1 expression in mouse tumors. **E** Immunohistochemical analysis of GIGYF1 and Ki-67 proteins in xenografts; Scale = 50 μm; * *P* < 0.05. In Figs. 1, 2, 3, 4, 5, 6, and 7, data are expressed as mean ± SD. In cell experiments, N = 3; in animal experiments, n = 5

To validate the effect of circPDK1 on PTX resistance, we analyzed the effect of PTX on mice treated with knockdown circPDK1 cells (H1299 cells). Xenograft mice with high circPDK1 expression exhibited a significant resistance phenotype to PTX treatment and shorter survival time compared to mice in the model group with knockdown circPDK1 (Fig. 7A). To further evaluate circPDK1 in NSCLC, tumor growth was observed in mice. PTX treatment can control NSCLC tumors in vivo (Fig. 7B, C). Further, RT-qPCR and Western Blot experiments showed that si-circPDK1 inhibited GIGYF1 expressions (Fig. 7D). Further, immunohistochemical analysis confirmed that GIGYF1 and Ki-67 were significantly decreased after suppressing circPDK1 (Fig. 7E).

## Discussion

Advanced NSCLC patients can benefit from chemotherapy (including PTX) [32], but efficacy of chemotherapy has been hampered by chemotherapy resistance [33]. Many circRNAs are involved in the regulation of tumorigenesis and chemotherapy resistance processes [34, 35], but the function of most circRNAs remains unclear. This study investigated circPDK1 competitively binding miR-4731-5p in positively regulating GIGYF1 expression in PTX resistance.

It is not only circRNA that is responsible for malignant tumor progression, but it is also believed to be associated with tumor drug resistance. Circ-0006528 regulates paclitaxel resistance in breast cancer [36], and circ-PVT1 can enhance PTX resistance of gastric cancer cells [37]. This study found a circRNA that is associated with drug resistance, and circPDK1 was highly expressed in NSCLC, which is consistent with the view that circPDK1 can promote lung cancer tumor growth confirmed by previous relevant literature. This study, like previous studies exploring circRNA and NSCLC drug resistance, not only validates circRNA as a marker for tumor diagnosis, but also reveals the possibility of addressing drug resistance. Currently circ-PVT1 is mostly used to study pancreatic cancer, renal cell carcinoma and colorectal cancer. Differently, this study demonstrates for the first time that circPDK1 is closely related to NSCLC, is a novel oncogene, and may have the potential to regulate drug resistance.

circRNA can sponge miRNA to function. This study found that circPDK1 negatively regulated miR-4731-5p which was poorly expressed in NSCLC. Increasing miR-4731-5p could improve PTX sensitivity and apoptotic rate, and inhibit proliferation and invasion of PTX-resistant cells, suggesting that circPDK1 targets miR-4731-5p to regulate PTX sensitivity. Combined with previous studies, miR-4731-5p is strongly associated with the prognosis of NSCLC patients, usually exhibiting distant metastasis, lymph node metastasis, and III/IV TNM stage. Based on these evidences, we inferred that miR-4731-5p could be a candidate biomarker for the early diagnosis of NSCLC, and that circPDK1/miR-4731-5p has a potential function as a relevant gene for NSCLC therapy.

circPDK1 can positively regulate GIGYF1 through miR-4731-5p. GIGYF1 was overexpressed in PTX-resistant NSCLC, promoting the anti-PTX properties of NSCLC cells, and GIGYF1 overexpression prevented miR-4731-5p upregulation-regulated PTX resistance in NSCLC cells. Finally, depleting circPDK1 in vivo inhibited GIGYF1, ultimately inhibiting the growth of drug-resistant tumors. Previously, GIGYF1 has been mostly used in studies on nerve damage and has not been involved in the field of cancer. Moreover, there are no relevant reports describing the antitumor effects of other miRNAs regulating GIGYF1 dysregulation, and we are the first to report miR-4731-5p-mediated modulation of GIGYF1 activity, especially in the context of NSCLC drug resistance. Thus, we identified a novel mechanism of circPDK1/miR-4731-5p/GIGYF1 pathway in this study, which may contribute to the treatment of NSCLC. What is more worthy of our deeper investigation is that GIGYF1 can bind to AKT to form a macromolecular complex, which plays an important role in regulating apoptosis and autophagy. When the expression level of GIGYF1 is inhibited, the expression level of p-AKT decreases, which leads to apoptosis [38, 39]. GIGYF1 can bind to the N-terminal region of Grb10, which promotes downstream ERK1/2 expression, thus facilitating cellular transcription and translation processes [40]. GIGYF1 may also indirectly regulate cell growth and proliferation. These speculative mechanisms suggest that GIGYF1 may regulate cell proliferation, apoptosis, transcription, and translation by binding to Grb10 or regulating the PI3K/Akt/mTOR signaling pathway. However, the exact regulatory mechanisms by which GIGYF1 regulates proliferation, apoptosis, and migration in PTX resistance still need to be followed up with in-depth investigation.

circPDK1 plays a key role in the onset and progression of NSCLC by targeting the expression of genes involved in the pathological progression of NSCLC to regulate cellular functions including cell proliferation, apoptosis, and metastasis, as well as resistance to therapy, and has great potential as a biomarker for early diagnosis and prognosis of NSCLC. In addition to this, the combination of circRNA inhibitors with PTX drugs may have enhanced sensitivity to chemotherapy. Adding circRNA to targeted therapy while reducing the dose of PTX drugs can significantly reduce dose-limiting adverse effects. circPDK1 can be applied to clinical therapy using a combination approach to develop more targeted and effective treatment strategies. However, tumor drug resistance is a multifactorial condition, and the complexity of the tumor microenvironment may give rise to variability in studies, making targeting circRNA to enhance chemosensitivity challenging and uncertain.

Although circPDK1 depletion inhibits tumor growth in vivo, but does not necessarily affect clinical outcomes, and the circRNA/miRNA/mRNAs regulatory network is very complex, so further research on the role of circPDK1 in clinical practice and the detailed physiological mechanisms are warranted. In addition, we did not clearly investigate the role of GIGYF1 in NSCLC chemoresistance with some limitations due to the insufficient sample size and selected cell lines. Next, we will further explore the function and role of circPDK1 in NSCLC in this direction and select more NSCLC cell lines for experiments to realize the reliability of the data.

In conclusion, suppressing circPDK1 inhibits proliferation and invasion of PTX-resistant cells, accelerates apoptosis, and acts as a molecular sponge of miR-4731-5p to downregulate GIGYF1, promote PTX sensitivity of NSCLC cells, and thus inhibit tumor growth in vivo. These results suggest that circPDK1 is a promising biomarker for predicting PTX response and that the circPDK1/miR-4731-5p/GIGYF1 axis may be a therapeutic target for NSCLC. This provides a new way to study tumor drug resistance and targeted therapy.

## Data Availability

The datasets used and/or analyzed during the present study are available from the corresponding author on reasonable request.
